# The Main Alkaloids in *Uncaria rhynchophylla* and Their Anti-Alzheimer’s Disease Mechanism Determined by a Network Pharmacology Approach

**DOI:** 10.3390/ijms22073612

**Published:** 2021-03-31

**Authors:** Peng Zeng, Xiao-Ming Wang, Chao-Yuan Ye, Hong-Fei Su, Qing Tian

**Affiliations:** Key Laboratory of Neurological Disease of National Education Ministry, School of Basic Medicine, Tongji Medical College, Huazhong University of Science and Technology, Wuhan 430030, China; zengp@hust.edu.cn (P.Z.); WangXiaoming@hust.edu.cn (X.-M.W.); M201875066@hust.edu.cn (C.-Y.Y.); Suhf@hust.edu.cn (H.-F.S.)

**Keywords:** Alzheimer’s disease, *Uncaria rhynchophylla*, alkaloids, network pharmacology, AD pathology

## Abstract

Alzheimer’s disease (AD) is a growing concern in modern society, and effective drugs for its treatment are lacking. *Uncaria rhynchophylla* (UR) and its main alkaloids have been studied to treat neurodegenerative diseases such as AD. This study aimed to uncover the key components and mechanism of the anti-AD effect of UR alkaloids through a network pharmacology approach. The analysis identified 10 alkaloids from UR based on HPLC that corresponded to 90 anti-AD targets. A potential alkaloid target-AD target network indicated that corynoxine, corynantheine, isorhynchophylline, dihydrocorynatheine, and isocorynoxeine are likely to become key components for AD treatment. KEGG pathway enrichment analysis revealed the Alzheimers disease (hsa05010) was the pathway most significantly enriched in alkaloids against AD. Further analysis revealed that 28 out of 90 targets were significantly correlated with Aβ and tau pathology. These targets were validated using a Gene Expression Omnibus (GEO) dataset. Molecular docking studies were carried out to verify the binding of corynoxine and corynantheine to core targets related to Aβ and tau pathology. In addition, the cholinergic synapse (hsa04725) and dopaminergic synapse (hsa04728) pathways were significantly enriched. Our findings indicate that UR alkaloids directly exert an AD treatment effect by acting on multiple pathological processes in AD.

## 1. Introduction

Alzheimer’s disease (AD), the most common form of dementia in the elderly, is characterized by the progressive deterioration of cognitive function and memory [1]. Approximately 50 million people were affected by AD worldwide in 2019, and nearly 10 million new cases occur every year [2]. The Chinese Center for Disease Control and Prevention estimated that there are more than 9 million AD patients in China at present, and it is expected that 40 million could be affected by 2050. AD not only brings a heavy mental burden and economic pressure to the family members and friends of patients but also places a very large burden on the social economy [3]. At present, the typical events in AD pathogenesis are considered to be the formation of extracellular amyloid-β (Aβ) plaques, intracellular accumulation of abnormally phosphorylated tau, neuronal synaptic dysfunction, and neuronal loss [4,5,6,7]. However, over the last decade, all clinical trials targeting a single target, such as Aβ or tau, have failed, and there are also no effective drugs for the prevention or treatment of AD in the clinic [8]. The anti-AD drugs currently approved by the Food and Drug Administration are cholinesterase inhibitors: donepezil, rivastigmine, tacrine, galantamine, and the N-methyl D-aspartate (NMDA) receptor antagonist memantine [9]. These drugs are symptomatic treatments and cannot halt the disease course. Moreover, the above drugs also cause a variety of unfavorable effects, including hypertensive crisis, nausea, diarrhea, and vomiting [10]. Given that multiple pathologies are associated with AD, the development of novel therapeutics that target these multiple pathologies is desirable in AD treatment. Therefore, the search for safer and effective drugs for the treatment of AD is extremely urgent.

Chinese herbal medicine has been widely used for dementia [11]. *Uncaria rhynchophylla* (UR), known as “*Gou-Teng*” in Chinese, has been used in traditional medicine in Asia, Africa and South America. Traditional Chinese medicine products or formulae containing UR have been used mainly to treat cardiovascular and central nervous system diseases, such as neurodegenerative diseases, lightheadedness, dizziness, convulsions, numbness, and hypertension [12,13,14,15]. Clinically, phytochemicals containing UR, such as Yigan-san (Yokukansan in Japanese), Tianmagouteng granules, and Gouteng-san, have become better herbal medicines for the treatment of stroke, hypertension, and chronic headache in China and Japan. The effects of UR are largely attributed to its predominant active component alkaloids. Therefore, the protective effects of UR and its main alkaloids on the central nervous system diseases have become a focus of research in recent decades.

UR is enriched in alkaloids, including rhynchophylline, isorhynchophylline, corynoxeine, isocorynoxeine, and hirsutine [16,17]. The total alkaloid content in UR is approximately 0.2%, among which rhynchophylline accounts for 28–50%, and isorhynchophylline accounts for 15% [18]. In 5xFAD mice, a transgenic mouse model of AD, UR ethanol extract (including rhynchophylline) significantly reduced Aβ aggregation and ameliorated AD-related pathologies (neuronal loss, synaptic degeneration, neuroinflammation, and neurogenesis) [19]. Rhynchophylline (bilateral hippocampal injection, 100 μM, 2 μL) was shown to improve soluble Aβ_1-42_-induced impairment of spatial cognition function by inhibiting excessive activation of extrasynaptic NR2B-containing NMDA receptors [20]. Treatment with isorhynchophylline (20 or 40 mg/kg/day) by gavage for 21 days improved Aβ_25-35_-induced cognitive impairment in rats *via* inhibition of neuronal apoptosis and tau protein hyperphosphorylation [21]. A recent study suggested that isorhynchophylline (20 or 40 mg/kg/day) treatment for 4 months improved cognitive impairment in TgCRND8 transgenic mice by reducing Aβ generation and deposition, tau hyperphosphorylation and neuroinflammation [22]. Moreover, 8 weeks of oral treatment with isorhynchophylline (20 or 40 mg/kg/day) could ameliorate D-galactose-induced learning and memory impairments by enhancing the antioxidant status and anti-inflammatory effect of D-galactose in brain tissues via NF-κB signaling [23]. Most of the mentioned studies focus on the therapeutic effects of UR alkaloids, only a few reports have shown that UR alkaloids can affect MAPT, GSK3B, AKT, BCL2, ect. Despite a series of studies on the biological activities of alkaloids in UR, the potential targets and underlying molecular mechanism of UR alkaloids in AD remain largely unclear. Due to the good curative effects of UR alkaloids on AD in animal researches and a variety of pathological processes involved, we therefore hypothesized that UR alkaloids possibly exert an AD treatment effect by acting on multiple pathological processes in AD.

**Figure 1:**
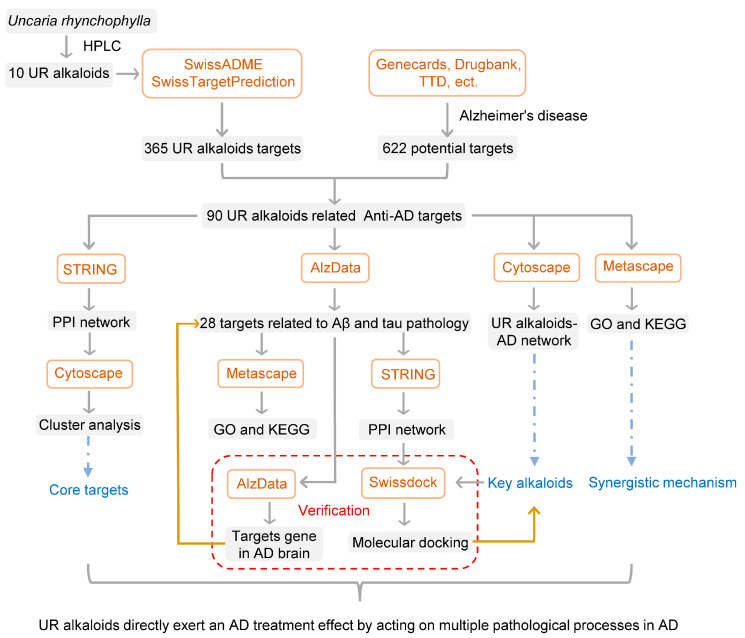
Flowchart of the study.

As an emerging approach for drug discovery, network pharmacology combines network biology and multipharmacology [24]. This study aimed to identify effective target proteins of UR alkaloids and uncover the mechanism of the anti-AD effect of UR alkaloids through a network pharmacology approach, microarray data analysis and molecular docking. Unlike other studies, we focus primarily on UR alkaloids, the predominant active ingredient of the UR. A scheme of the study protocol is shown in Figure 1. We obtained UR alkaloids from a recent study based on high-performance liquid chromatography (HPLC) [17], this increases the quality of this study significantly. In addition to enrichment analyses, an AD database was used to analyze the correlation between the targets of UR alkaloids against AD and AD pathology in this study. Furthermore, we also used the human high-throughput omics data to validate the targets of UR alkaloids against AD. Our study offers new insight into the mechanisms of UR alkaloids and provides a more specific and effective treatment for AD.

## 2. Results

### 2.1. The Chemical Structure and ADME Properties of the Main Alkaloids from UR

**Figure 2:**
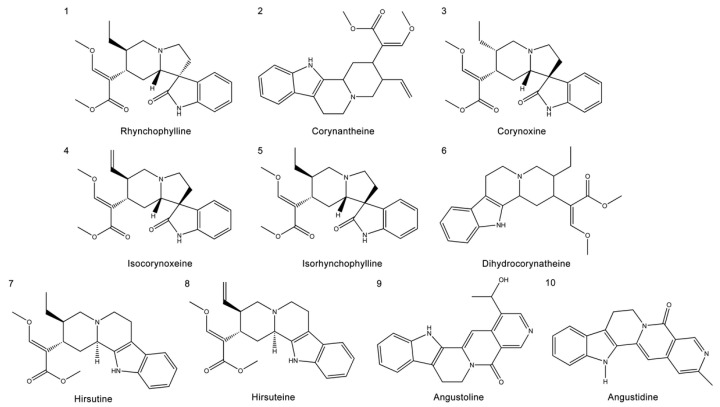
Structures of the main alkaloids extracted from *Uncaria rhynchophylla* (UR).

This study primarily focused on the therapeutic effect of UR alkaloids against AD. A recent study identified 10 alkaloids from UR based on HPLC [17]. The extraction rate of the UR alkaloids (100 g) was 9.4%. The chemical structures of the main alkaloids were obtained from the PubChem database and are shown in Figure 2. Next, we used an online tool SwissADME for an in-depth evaluation of the ADME-related properties of the main alkaloids from UR. SwissADME prediction showed that all the main alkaloids satisfied Lipinski’s rule of five, and other chemical and pharmacological properties, including topological polar surface area (TPSA) and solubility (LogS), were also evaluated (Table 1). These results indicate that these alkaloids may exhibit good permeability across cell membranes.

### 2.2. Screening of Targets of the Main Alkaloids from UR in AD

**Figure 3:**
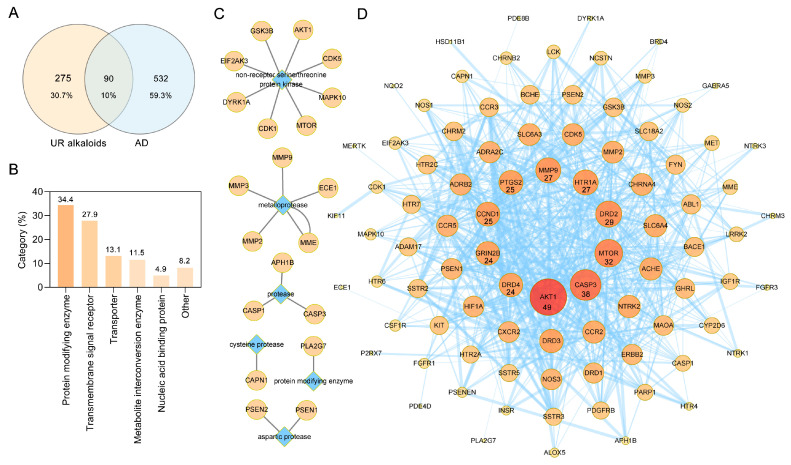
Protein–protein interaction (PPI) network construction for target proteins of UR alkaloids against Alzheimer’s disease (AD). (**A**) A Venn diagram was applied to obtain the intersection between the 10 UR alkaloids and AD targets. (**B**) Panther classification was used categorize common targets of UR alkaloids against AD. (**C**) Target proteins involved in protein modification as enzyme (PC00260). (**D**) PPI network of UR alkaloids against AD. Nodes represent target proteins, and edges represent interactions among targets. The numbers below the nodes indicate the degree. The darker the color and the larger the node are, the greater the degree is.

Potential targets of the 10 alkaloids were predicted via the SwissTargetPrediction database based on their structure, and a total of 365 targets were obtained. Results from the GeneCards, DrugBank, Therapeutic Target Database (TTD), and Chemogenomics Database for Alzheimer’s Disease identified a total of 622 targets relevant to AD. Ultimately, a Venn diagram was used to summarize 90 common targets associated with both UR alkaloids and AD for further analysis (Figure 3A). Detailed information about these common targets is provided in Table 2. Furthermore, these 90 target proteins of UR alkaloids against AD were categorized into 6 different classes based on their cellular functions, of which protein-modifying enzyme (PC00260, 34.4%) was the most enriched class (Figure 3B). Among these protein-modifying enzymes, AKT1, CDK1, CDK5, EIF2AK3, GSK3B, MAPK10, MTOR and DYRK1A are nonreceptor serine/threonine protein kinases, and ECE1, MME, MMP2, MMP3 and MMP9 are metalloproteases (Figure 3C). The above results indicated that alkaloids from UR can protect against AD through multiple targets and biological functions.

### 2.3. PPI Analysis of Targets of the Main Alkaloids against AD

To explore the relationship between these 90 targets of alkaloids against AD, protein–protein interaction (PPI) analysis was performed using the STRING database [25]. A PPI network with a total of 90 nodes and 610 edges and an average node degree of 13.6 was generated (Figure 3D). The darker the color and the larger the node were, the greater the degree was. AKT1, CASP3, MTOR, DRD2, HTR1A, MMP9, PTGS2, CCND1, GRIN2B, and DRD4, which are ranked by degree, were identified as core targets (Figure 3D). Among these, AKT1 showed the highest degree (49). This demonstrated that these core targets are closely related to other targets in the PPI network, suggesting that these targets may play important roles in AD treatment.

### 2.4. Clusters of Common Targets of the Main Alkaloids against AD

**Figure 4:**
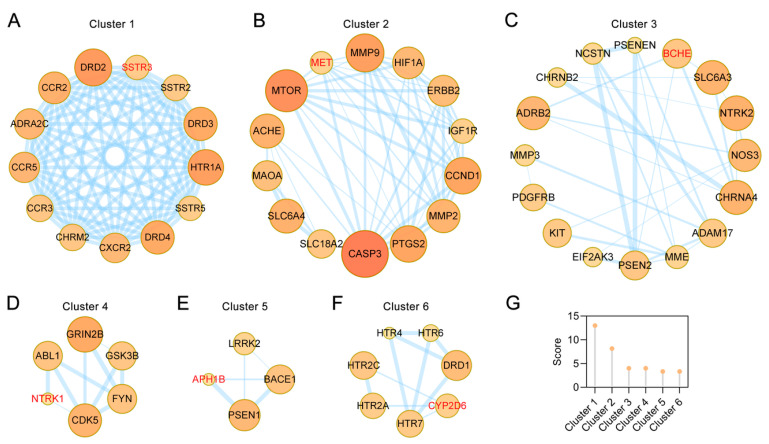
PPI network clusters of common targets of both UR alkaloids and in AD. (**A**–**F**) Clusters 1 to 6 were found with Molecular Complex Detection (MCODE), which can identify densely connected regions. The seed node of each cluster is indicated by red font. (**G**) Comparison of the MCODE scores of different clusters.

Six clusters were found after the PPI network of the main alkaloids against AD was analyzed through Molecular Complex Detection (MCODE) (k-core = 2). This demonstrates that these clusters may be the most relevant to AD treatment. The details are provided in Figure 4. Cluster 1 contains 13 nodes and 78 edges with a score of 13. The seed node of this cluster is somatostatin receptor 3 (SSTR3), which regulates diverse cellular functions, such as neurotransmission, cell proliferation, and endocrine signaling (Figure 4A). Cluster 2 contains 14 nodes and 53 edges with a score of 8.15. The seed node of this cluster is MET proto-oncogene receptor tyrosine kinase (MET), which encodes a member of the receptor tyrosine kinase family of proteins and the product of the proto-oncogene MET (Figure 4B). Cluster 3 contains 16 nodes and 30 edges with a score of 4. The seed node of this cluster is butyrylcholinesterase (BCHE), which contributes to inactivation of the neurotransmitter acetylcholine and degrades neurotoxic organophosphate esters (Figure 4C). Cluster 4 contains 6 nodes and 10 edges with a score of 4. The seed node of this cluster is neurotrophic receptor tyrosine kinase 1 (NTRK1) (Figure 4D). Cluster 5 contains 4 nodes and 5 edges with a score of 3.33. The seed node of this cluster is aph-1 homolog B (APH1B) (Figure 4E). Cluster 6 contains 7 nodes and 10 edges with a score of 3.33. The seed node of this cluster is cytochrome P450 family 2 subfamily D member 6 (CYP2D6) (Figure 4F). The respective MCODE scores are listed in Figure 4G.

### 2.5. Construction of a Main Potential Alkaloid Target-AD Target Network

**Figure 5:**
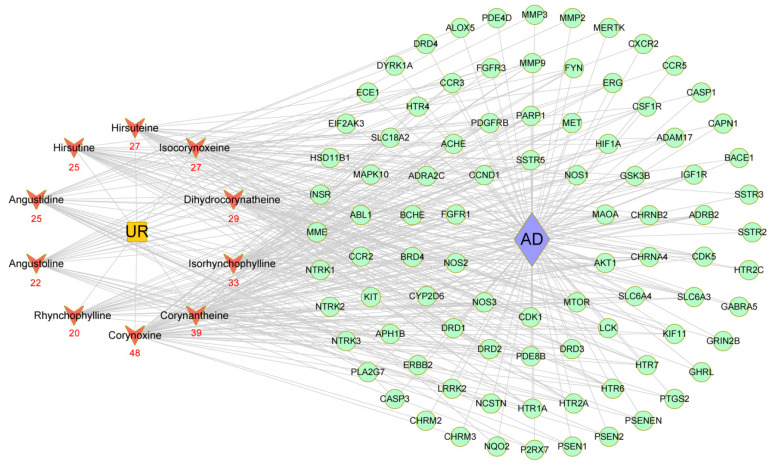
Potential alkaloid target-AD target network. Red nodes represent the main alkaloids extracted from UR, and the numbers beneath each node represent the number of UR targets against AD. Green nodes represent shared targets between potential targets of UR alkaloids and AD targets. UR: *Uncaria rhynchophylla*; AD: Alzheimer’s disease.

To explore the therapeutic mechanism of the main alkaloids in the treatment of AD, 90 common targets and 10 main alkaloids from UR were used to construct a main potential alkaloid target-AD target network (Figure 5). All of these alkaloids are related to multiple targets, resulting in 295 component-target associations between 10 alkaloids and 90 targets. The average number of targets per alkaloid was 29.5, and the mean degree of components per target was 3.3. This clearly indicates that UR fits the multicomponent and multitarget characteristics of traditional Chinese medicine. Corynoxine (degree = 48) had the most targets, followed by corynantheine (degree = 39), isorhynchophylline (degree = 33), dihydrocorynatheine (degree = 29), isocorynoxeine (degree = 27), and hirsuteine (degree = 27), indicating that these alkaloids from UR are highly likely to become key components in AD treatment.

### 2.6. Potential Synergistic Mechanism of the 10 Alkaloids against AD

#### 2.6.1. Gene Ontology (GO) Enrichment Analysis

**Figure 6:**
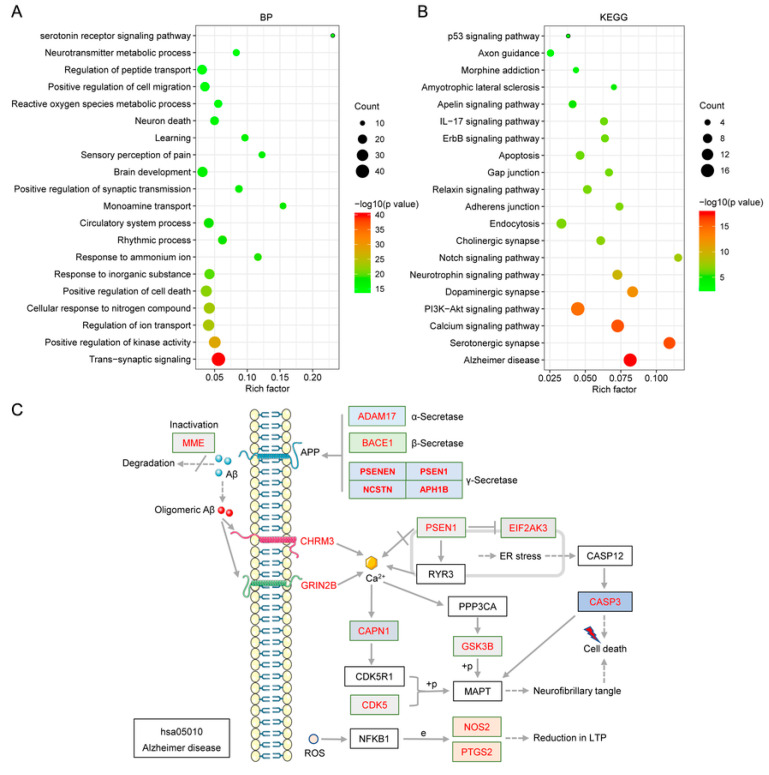
GO biological process (BP) (**A**) terms and the results of KEGG (**B**) pathway enrichment analysis of target proteins of UR alkaloids against AD. The X-axis represents the rich factor, the bubble size represents the number of targets enriched in terms, and the color indicates the *p*-value. (**C**) Schematic drawing of the Alzheimer disease pathway (hsa05010). Red font indicates the targets of UR alkaloids involved in the Alzheimer disease pathway.

The common targets between UR alkaloids and AD were further analyzed for functional prediction by Metascape. The primary enriched GO biological process (BP) terms were transsynaptic signaling (GO:0099537), positive regulation of kinase activity (GO:0033674), regulation of ion transport (GO:0043269), cellular response to nitrogen compound (GO:1901699), positive regulation of cell death (GO:0010942), response to inorganic substance (GO:0010035), and response to ammonium ion (GO:0060359) (Figure 6A). In particular, 7 out of 10 targets involved in transsynaptic signaling were core targets (AKT1, DRD2, DRD4, GRIN2B, HTR1A, MTOR, and PTGS2). These findings implicated the involvement of multiple targets and various biological processes in the multiple synergistic effects of UR alkaloids against AD.

#### 2.6.2. Pathway Analysis to Explore the Therapeutic Mechanisms of the Main Alkaloids in AD

**Figure 7:**
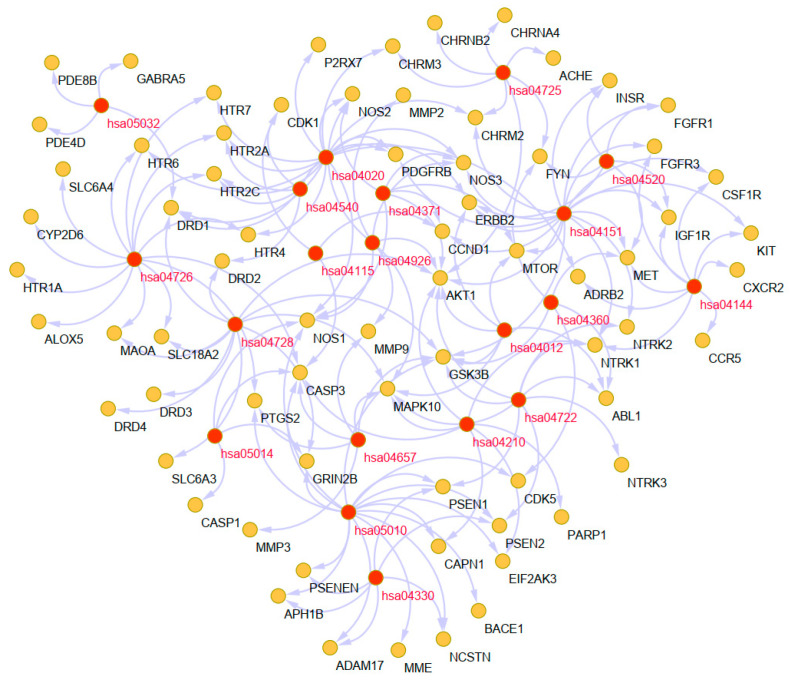
Target-enriched KEGG pathway network for UR alkaloids against AD. Red nodes represent enriched KEGG pathways, and brown nodes represent target proteins.

The Kyoto Encyclopedia of Genes and Genomes (KEGG) pathway database is a collection of manually drawn pathway maps of molecular interactions. To identify signaling pathways associated with targets of the main alkaloids against AD, Metascape was used to identify pathways enriched in these 90 targets. The involved KEGG pathways were mainly the Alzheimer disease (hsa05010), serotonergic synapse (hsa04726), the calcium signaling pathway (hsa04020), the PI3K-Akt signaling pathway (hsa04151), dopaminergic synapse (hsa04728), the neurotrophin signaling pathway (hsa04722), the notch signaling pathway (hsa04330), and cholinergic synapse (hsa04725) (Figure 6B). Detailed information on the KEGG pathway enrichment analysis is shown in Table 3. The most significantly enriched pathway, the Alzheimer disease pathway (hsa05010, *p* = 2.4E-18), belongs to the Human Diseases category pathway, and is mainly related to the production and clearance of Aβ and aberrant tau hyperphosphorylation (Figure 6C). A network of the targets and pathways enriched in the 10 UR alkaloids against AD is shown in Figure 7 and contains 90 nodes, including the top 20 KEGG pathways associated with 70 targets and 163 edges. Based on the results of pathway analysis, it was found that these pathways are closely related to AD, axons, and synapses. In particular, the PI3K-Akt signaling pathway (hsa04151) exhibits the highest number of target connections (degree = 17), and its targets include AKT1, CCND1, ERBB2, MTOR, GSK3B, MET, NOS3, and PDGFRB. Next, the Alzheimer disease pathway (hsa05010) exhibits the second largest number of degrees (16), and the associated targets include ADAM17, BACE1, CASP3, CDK5, GSK3B, PSEN1, PSEN2, PSENEN, and NOS1.

### 2.7. Bioinformatics Analysis of Targets of the Alkaloids Related to Aβ and Tau Pathology

**Figure 8:**
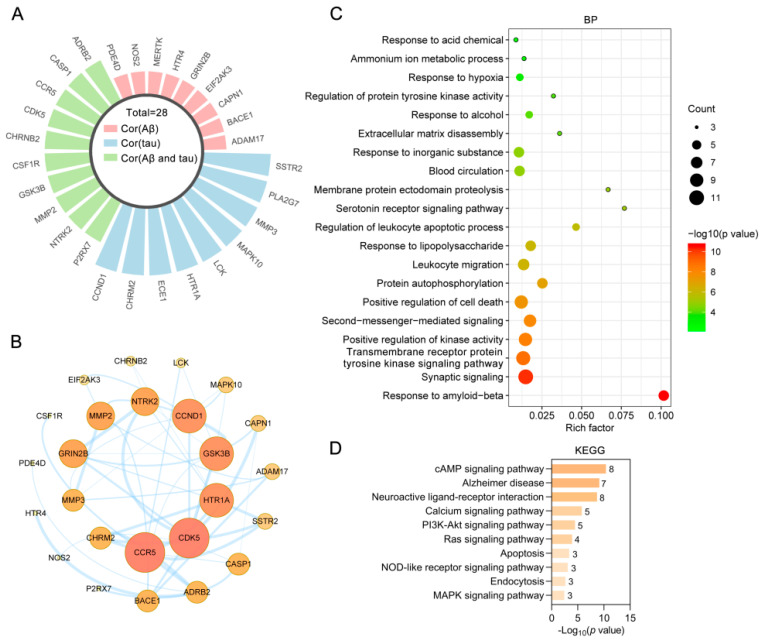
Bioinformatics analysis of targets of UR alkaloids related to Aβ and tau pathology. (**A**) Radial bar plot showing targets of the alkaloids significantly correlated with tau, Aβ, or Aβ and tau. (**B**) PPI network of proteins associated with the pathology of Aβ and tau. The darker the color and the larger the node are, the higher the degree is. (**C**) Bubble chart of the top 20 biological process (BP) terms identified by GO enrichment analysis. The X-axis represents the rich factor, the bubble size represents the number of targets enriched in terms, and the color indicates the *p*-value. (**D**) KEGG pathway enrichment analysis of targets correlated with tau, Aβ, or Aβ and tau. Numbers next to bar graphs indicate the numbers of targets enriched in the terms.

To further verify the relationship between the potential targets of the alkaloids and Aβ and tau pathology, the AlzData database [26] was used. Among the targets, 28 out of 90 were significantly correlated with tau, Aβ, or Aβ and tau (Figure 8A). Specifically, ADAM17, BACE1, CAPN1, EIF2AK3, GRIN2B, HTR4, MERTK, NOS2, and PDE4D were significantly correlated with Aβ pathology; CCND1, CHRM2, ECE1, HTR1A, and so on were significantly correlated with tau pathology; and ADRB2, CASP1, CCR5, CDK5, CHRNB2, CSF1R, GSK3B, MMP2, NTRK2, and P2RX7 were significantly associated with both Aβ and tau pathology. To construct a PPI network, the gene IDs of the 28 targets were input into the STRING database. A total of 25 nodes and 45 edges were found in the PPI network, and CCR5, CDK5, HTR1A, GSK3B, CCND1, NTRK2, MMP2, GRIN2B, MMP3, and CHRM2 were identified as core targets ranked by degree (Figure 8B).

**Figure 9:**
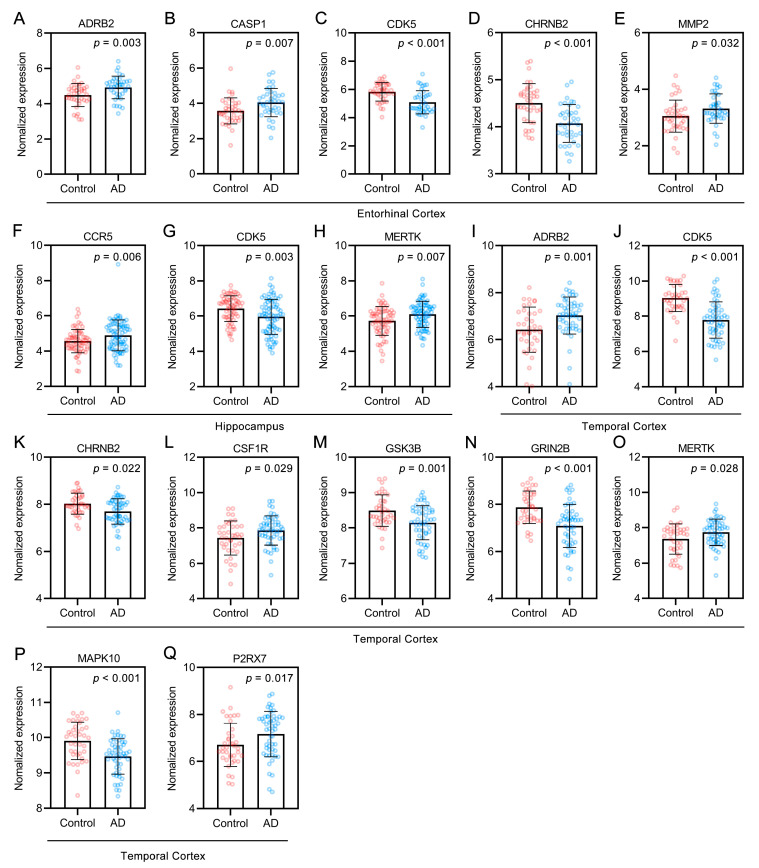
(**A**–**Q**) Targets of UR alkaloids against AD in the control and AD groups of the GEO dataset. Entorhinal cortex, *n* = 39 in each group. Hippocampus, *n* = 66 in the healthy control group, *n* = 74 in the AD group. Temporal cortex, *n* = 39 in the healthy control group, *n* = 52 in the AD group. Values are presented as the mean ± SD.

The 28 targets were further analyzed for functional prediction by Metascape. GO BP terms primarily enriched in the targets included the response to amyloid-beta (GO:1904645), synaptic signaling (GO:0099536), the transmembrane receptor protein tyrosine kinase signaling pathway (GO:0007169), positive regulation of kinase activity (GO:0033674), second messenger-mediated signaling (GO:0019932), and positive regulation of cell death (GO:0010942) (Figure 8C). In particular, synaptic signaling (GO:0099536) exhibited the highest number of target connections (degree = 11), and the targets included ADRB2, BACE1, CDK5, CHRM2, CHRNB2, GRIN2B, GSK3B, HTR1A, HTR4, NTRK2, and P2RX7. In the KEGG pathway analysis, targets related to the MAPK signaling pathway (hsa04010), endocytosis (hsa04144), the NOD-like receptor signaling pathway (hsa04621), apoptosis (hsa04210), the Ras signaling pathway (hsa04014), and the PI3K-Akt signaling pathway (hsa04151) were highly enriched (Figure 8D). ADAM17, BACE1, CAPN1, CDK5, EIF2AK3, GRIN2B, and GSK3B were enriched in the Alzheimer disease pathway (hsa05010). Normalized expression targets of the main alkaloids against AD in the control and AD groups of the Gene Expression Omnibus (GEO) dataset were analyzed by the “Differential expression” module of the AlzData database (Figure 9). Of the targets, ADRB2, CASP1, CCR5, CSF1R, MERTK, MMP2, and P2RX7 were significantly upregulated, and CDK5, CHRNB2, GRIN2B, GSK3B, and MAPK10 were significantly downregulated in AD patients compared to controls. The above results indicated that the alkaloid targets are intimately associated with the pathology of Aβ and tau.

### 2.8. Molecular Docking Simulation

From the main potential alkaloid target-AD target network (Figure 5), corynoxine (degree = 48) and corynantheine (degree = 39) had the highest number of targets against AD. Therefore, molecular docking analysis was used to validate the binding of corynoxine and corynantheine to core targets related to AD pathology. Delta G is defined as the binding energy based on the ensemble free energy; the greater the absolute value of Delta G is, the more stable binding is. The results of molecular docking of corynoxine and corynantheine to core targets are shown in Table 4. Corynoxine and corynantheine exhibited strong binding to all core targets. Among these targets, corynoxine showed the highest binding energy with CCR5, MMP3, CCND1, and NTRK2, with delta G values of −9.28, −8.52, −8.17 and −8.10 kcal/mol, respectively; corynantheine showed the highest binding energy with CHRM2, MMP3, MMP2, GRIN2B, with delta G values of −9.38, −9.30, −9.24, and −9.02 kcal/mol, respectively.

## 3. Discussion

As the predominant active pharmacological components in UR, UR alkaloids have been widely used as an intervention for neurodegenerative diseases in animal models for several years [12,15,17,19,20,21]. UR alkaloids are mainly administered by oral administration of monomers in the experiments above mentioned. Unfortunately, the potential targets UR alkaloids are not clear at present. This study comprehensively investigated the therapeutic effect of UR alkaloids on AD.

Generally, network pharmacology studies use public databases to obtain the main components in traditional Chinese Medicine. Conventional screening methods of public databases do not take into account the content and distribution specificity of components, which will lead to a large number of non-specific components to be screened out. For instance, beta-sitosterol exists widely in 567 kinds of herbs and stigmasterol 284 kinds of herbs (data from HERB, http://herb.ac.cn/ (accessed on 30 March 2021)). UR alkaloids have significant pharmacological activities and are also representative active ingredients in UR. Accordingly, alkaloids in UR were obtained by HPLC based on a recent study [17], this greatly improved the quality of the data collected. In the present study, all of the 10 alkaloids in UR were in agreement with Lipinski’s rule of five, demonstrating that the alkaloids have acceptable pharmacokinetic properties. Furthermore, 90 potential targets of these 10 alkaloids against AD were obtained using network pharmacology strategies. A potential alkaloid target-AD target network indicated that corynoxine, corynantheine, isorhynchophylline, dihydrocorynatheine, isocorynoxeine, and hirsuteine are likely to become key components for AD treatment. Isorhynchophylline, isocorynoxeine, and hirsuteine showed high permeability, with apparent permeability coefficient values for bidirectional transport at the 10^−5^ cm/s level [27]. Corynoxine, a natural neuroprotective autophagy enhancer [28], has the greatest number of therapeutic targets for AD (degree = 48). Corynoxine can regulate several kinases, including RPS6KB1, MAP2K2, and PLK1, contributing to the clearance of AD-associated β-amyloid precursor protein (APP) and Parkinson disease-associated α-synuclein [29]. The molecular docking results also showed that corynoxine strongly binds CCR5, MMP3, CCND1, and NTRK2. Treatment with isorhynchophylline (20 or 40 mg/kg/day) could ameliorate Aβ_25-35_- or D-galactose-induced cognitive impairment in animal models [21,23]. Furthermore, in vitro experiments demonstrated that isorhynchophylline and rhynchophylline (pretreating 100 μM for 2 h) significantly decreased Aβ-induced cell death, intracellular calcium overload, and tau protein hyperphosphorylation in PC12 cells [30]. Notably, there have been few studies on the treatment of AD with corynantheine and dihydrocorynatheine until now. For future research, more attention might be paid to these alkaloids in the treatment of AD.

Senile plaques (SPs), neurofibrillary tangles (NFTs), neuronal synaptic dysfunction and neuronal loss are characteristic pathologic changes in AD [7,31]. KEGG pathway enrichment analysis revealed that the Alzheimer disease pathway (hsa05010) was the most significantly enriched pathway in UR alkaloids against AD. Among the associated targets, ADAM17 (an α-secretase), BACE1 (a β-secretase), APH1B, NCSTN, PSEN1, and PSENEN (a γ-secretase) are involved in APP cleavage. In AD brains, ADAM17-positive neurons often colocalize with amyloid plaques and are considered potential therapeutic targets for AD [32]. Moreover, ADAM17 is also involved in the cleavage of many other membrane-bound proteins, especially some inflammatory factors related to microglial activation [33]. BACE1 is a rate-limiting enzyme for Aβ production. The inhibition of BACE1 activity could block one of the earliest pathologic events in AD. Several BACE1 inhibitor drug candidates have advanced to phase 3 clinical trials [34]. γ-Secretase activity is mediated by a multiprotein complex consisting of Presenilin, APH1, PEN2 and Nicastrin (NCT) [35]. PSEN1 mutations are responsible for the majority of familial AD cases, and more than 300 mutations in PSEN1 have been reported [36]. Thus, UR alkaloids could decrease Aβ generation and deposition in the brain through several important drug targets. In addition, targets for regulating tau phosphorylation, such as CAPN1, CDK5 and GSK3B, are involved in the anti-AD effects of the UR alkaloids. CDK5 and GSK3B belong to the nonreceptor serine/threonine protein kinase family. GSK3B is a major tau kinase involved in the development of AD tau pathology [37]. A previous study showed that Aβ accumulation in the AD brain can activate kinases that promote tau phosphorylation, including GSK3B [38]. Interestingly, apart from the targets related to the Alzheimer’s disease pathway, up to 28 out of 90 targets were significantly correlated with tau, Aβ, or Aβ and tau. Among these targets, the response to amyloid-beta (GO:1904645) was the most significantly enriched, and the synaptic signaling term (GO:0099536) exhibited the highest number of target connections. CCR5, a cytokine belonging to the β chemokine receptor family of integral membrane proteins, is significantly positively associated with both Aβ and tau pathology. CCR5 expression is strongly related to microglia and inflammation, which accelerate the development of AD [39]. Of the 28 targets related to Aβ and tau pathology, CCR5 had the highest degree in the PPI network and was significantly increased in the hippocampus of AD patients. We should pay more attention to CCR5 in future studies. In addition to affecting Aβ and tau pathology, a series of synaptic-related KEGG pathways, such as serotonergic synapses (hsa04726), dopaminergic synapses (hsa04728), and cholinergic synapses (hsa04725), were also enriched. Cholinergic synapses are ubiquitous in the human central nervous system. The cholinergic hypothesis of AD centers on the progressive loss of limbic and neocortical cholinergic innervation [40]. Cholinesterase inhibitors increase the availability of acetylcholine at synapses in the brain and are one of the few drug therapies that have been proven clinically useful in the treatment of AD, thus validating the cholinergic system as an important therapeutic target in this disease. Overall, our findings indicate that UR alkaloids can directly treat AD by acting on multiple pathological processes in AD.

Akt is a serine/threonine protein kinase and an important kinase downstream of PI3K. There are three AKT isoforms (AKT1-3), among which AKT1 is the most important subtype. In this study, the PI3K-Akt signaling pathway (hsa04151) was found by KEGG pathway enrichment analysis to be an important pathway by which UR alkaloids counteract AD, and 17 targets are involved (including the core targets AKT1, CCND1, and MTOR). Among signal transduction pathways, this pathway is a key mediator that regulates cell growth, inflammation, metabolism, and cell survival in response to growth factors. Previous studies have found that Aβ oligomers inhibit the PI3K-Akt pathway, which leads to neuronal death [41]. In the AD brain, normalized pT308 AKT1 was positively correlated with both the amyloid burden and tau tangle density [42]. The PI3K-Akt signaling pathway plays an important role in GSK3B activity regulation, as AKT promotes the phosphorylation of GSK3B, resulting in GSK3B inactivation [43,44]. Furthermore, the core target CASPS (degree = 38) was also involved in AD treatment. CASP3 is a pivotal executioner of apoptosis that plays a major role in neuronal death during nervous system development and under certain pathological conditions. Neuronal cell injury and loss are major neuropathological features of AD. The level of activated CASP3 was elevated in the brains of patients with severe definitive AD [45]. The positive regulation of cell death (GO:0010942) and neuron death (GO:0070997) were also significantly enriched in the present study.

A total of 10 alkaloids in UR and 90 common targets against AD were screened by network pharmacology analysis. GO enrichment and KEGG pathway enrichment analyses suggested that UR alkaloids can directly treat AD by acting on multiple AD pathological processes, such as processes involving Aβ and tau, neuronal synaptic function, and neuronal loss. However, clinical studies are necessary to verify these theoretical observations in the future. The bioavailability of UR alkaloids after oral administration was not reported in the clinic. The oral bioavailability of rhynchophylline, isorhynchophylline, hirsutine, and angustidine was 41.82%, 12.71%, 34.44%, and 51.85%, respectively, based on the Traditional Chinese Medicine Systems Pharmacology Database and Analysis Platform (TCMSP, https://tcmspw.com/tcmsp.php (accessed on 30 March 2021)). It is necessary to further optimize the structure or improve the dosage form to increase the oral bioavailability for its clinical applicability. Taken together, our findings might provide a theoretical basis for the use of UR alkaloids as a therapeutic for AD.

## 4. Materials and Methods

### 4.1. Collection of the Main Alkaloids from UR and ADME Evaluation

The main alkaloids in UR were obtained from a recent study based on HPLC [17]. Lipinski’s rule of five was used to assess the in vivo absorption abilities of the designed compounds [46,47]. Compounds that meet the requirements of Lipinski’s rule of five seem to be more likely to become a drug. Specifically, Lipinski’s rule of five includes the following: a molecule weight (MW) < 500, number of hydrogen bond donors (Hdon) ≤ 5, number of hydrogen bond acceptors (Hacc) ≤ 10, lipid–water partition coefficient (LogP) ≤ 5 and number of rotatable bonds (Rbon) ≤ 10. The SwissADME web tool (http://www.swissadme.ch (accessed on 30 March 2021)) [48,49] was used to evaluate the ADME of the compounds. Moreover, the TPSA, LogS, and skin permeation (Log Kp) were also measured.

### 4.2. Target Fishing

#### 4.2.1. Collection of the Main Alkaloid-Related Targets

All chemical structures and canonical SMILES strings for the main alkaloids were obtained from the PubChem database (https://pubchem.ncbi.nlm.nih.gov/ (accessed on 30 March 2021)) [50]. To obtain the targets of the main alkaloids in UR, SwissTargetPrediction (http://www.swisstargetprediction.ch/ (accessed on 30 March 2021)) [51] was employed.

#### 4.2.2. Screening of Targets of the Main Alkaloids against AD

Disease targets were collected from the GeneCards database (https://www.genecards.org/ (accessed on 30 March 2021)), DrugBank database (https://go.drugbank.com/ (accessed on 30 March 2021)) [52], TTD (http://bidd.nus.edu.sg/group/cjttd/ (accessed on 30 March 2021)) [53], and Chemogenomics Database for Alzheimer’s Disease (https://www.cbligand.org/AD/mainpage.php (accessed on 30 March 2021)) using “Alzheimer’s disease” as a key phrase, and duplicate targets were removed using Microsoft Excel software. The intersection of UR alkaloid-related targets and AD-related targets was assessed by Venny 2.1 (https://bioinfogp.cnb.csic.es/tools/venny/index.html (accessed on 30 March 2021)). Common targets represent the target of UR alkaloids against AD. In addition, protein classification was performed using the Panther classification system (http://pantherdb.org/ (accessed on 30 March 2021)) [54].

### 4.3. PPI Network Construction and Clustering Analysis

A PPI network was constructed by using the STRING database version 11.0 (https://string-db.org/ (accessed on 30 March 2021)) [25]. The organism was set to *Homo sapiens*, and only the minimum required interaction score > 0.4 was chosen as significant. PPI networks consist of nodes, which represent a target protein, and edges, which represent protein–protein interactions. The thickness of an edge represents the combined score. Degree refers to the number of other nodes directly connected to a node. The higher the degree is, the more important the node is. Core targets were identified through network analysis using Cytoscape software (v.3.7.1) [55] and its plugin (Network Analysis). In the present study, the top 10 proteins ranked by degree were selected and identified as core targets.

The Cytoscape plugin MCODE [56] was applied to analyze clustering modules in the PPI network. The MCODE criteria for selection were as follows: degree cutoff = 2, node score cutoff = 0.2, k-core = 2, and max depth = 100. A node with the highest weighted vertex was defined as a seed node, the key target of this cluster, by MCODE. Moreover, a potential alkaloid target-AD target network was constructed using Cytoscape software.

### 4.4. GO and KEGG Pathway Enrichment Analyses

Metascape (https://metascape.org/gp (accessed on 30 March 2021)) [57] is a comprehensive tool for gene annotation and enrichment analysis. GO biological process and KEGG pathway enrichment analyses were performed using Metascape. The enriched terms with *p* < 0.01, a minimum count of 3, and an enrichment factor > 1.5 were considered significant. The top 20 enriched terms were visualized using an online tool (www.bioinformatics.com.cn (accessed on 30 March 2021)). A target-enriched KEGG pathway network for the main alkaloids against AD was also constructed by Cytoscape software. Red nodes represent enriched KEGG pathways, and brown nodes represent target proteins.

### 4.5. Analysis of Alkaloid Targets Related to AD Pathology

AlzData (http://www.alzdata.org/ (accessed on 30 March 2021)) [26] is an AD database that collects current high-throughput omics data. Gene symbols for the target proteins of UR alkaloids against AD were given as input to the AlzData database for correlation analysis of AD pathology (Aβ and tau). Microsoft Excel was then used to collate the obtained results. The targets of the UR alkaloids related to AD pathology were used to conduct further GO and KEGG pathway enrichment analyses. Normalized expression targets of alkaloids against AD in the control and AD groups of the GEO dataset were analyzed by the “Differential expression” module of AlzData. GraphPad Prism software was used for graphical visualization. Values are presented as the mean ± SD.

### 4.6. Molecular Docking

To validate the binding of UR alkaloids on predicted core targets, the 3D molecular structure of UR alkaloids was retrieved from the PubChem database and the structure files of target proteins were acquired from the RCSB Protein Data Bank (PDB database, http://www.rcsb.org/ (accessed on 30 March 2021)) [58]. Molecular docking calculations were performed using the SwissDock web service (http://www.swissdock.ch/docking (accessed on 30 March 2021)) [59].

## 5. Conclusions

In this study, a network-based computational strategy and molecular docking were used to uncover the pharmacological mechanism of the UR alkaloids on AD. A total of 10 alkaloids in UR and 90 common targets against AD were screened by network pharmacology. GO enrichment analysis and KEGG pathway enrichment analysis suggested that UR alkaloids exerted the effect of treating AD directly act on multiple AD pathological processes such as Aβ overproduction, tau hyperphosphorylation, synaptic dysfunction, and neuronal loss. Taken together, our findings might provide a theoretical basis for the use of UR alkaloids as a therapeutic for AD.

## Figures and Tables

**Table 1:** Pharmacological and molecular properties of the main alkaloids in UR.

Name	Formula	MW (g/mol)	Hdon	Hacc	Rbon	TPSA (Å)	LogP	LogS	Log Kp (cm/s)
Rhynchophylline	C_22_H_28_N_2_O_4_	384.47	1	5	5	67.87	2.43	−3.51	−7.01
Corynantheine	C_22_H_26_N_2_O_3_	366.45	1	4	5	54.56	3.08	−4.03	−6.28
Corynoxine	C_22_H_28_N_2_O_4_	384.47	1	5	5	67.87	2.38	−3.51	−7.01
Isocorynoxeine	C_22_H_26_N_2_O_4_	382.45	1	5	5	67.87	2.26	−3.34	−7.17
Isorhynchophylline	C_22_H_28_N_2_O_4_	384.47	1	5	5	67.87	2.4	−3.51	−7.01
Dihydrocorynatheine	C_22_H_28_N_2_O_3_	368.47	1	4	5	54.56	3.22	−4.21	−6.11
Hirsutine	C_22_H_28_N_2_O_3_	368.47	1	4	5	54.56	3.22	−4.21	−6.11
Hirsuteine	C_22_H_26_N_2_O_3_	366.45	1	4	5	54.56	3.1	−4.03	−6.28
Angustoline	C_20_H_17_N_3_O_2_	331.37	2	3	1	70.91	2.52	−3.52	−7.05
Angustidine	C_19_H_15_N_3_O	301.34	1	2	0	50.68	3.02	−4.01	−6.24

MW: molecule weight; Hdon: hydrogen bond donors; Hacc: hydrogen bond acceptors; Rbon: rotatable bonds; TPSA: topological polar surface area; LogP: lipid–water partition coefficient; LogS: solubility; Log Kp: skin permeation.

**Table 2:** Targets of UR alkaloids against AD.

Number	Gene ID	Gene Symbol	Description
1	25	ABL1	ABL proto-oncogene 1, non-receptor tyrosine kinase
2	43	ACHE	acetylcholinesterase (Cartwright blood group)
3	6868	ADAM17	ADAM metallopeptidase domain 17
4	152	ADRA2C	adrenoceptor alpha 2C
5	154	ADRB2	adrenoceptor beta 2
6	207	AKT1	AKT serine/threonine kinase 1
7	240	ALOX5	arachidonate 5-lipoxygenase
8	83464	APH1B	aph-1 homolog B, gamma-secretase subunit
9	23621	BACE1	beta-secretase 1
10	590	BCHE	butyrylcholinesterase
11	23476	BRD4	bromodomain containing 4
12	823	CAPN1	calpain 1
13	834	CASP1	caspase 1
14	836	CASP3	caspase 3
15	595	CCND1	cyclin D1
16	729230	CCR2	C-C motif chemokine receptor 2
17	1232	CCR3	C-C motif chemokine receptor 3
18	1234	CCR5	C-C motif chemokine receptor 5
19	983	CDK1	cyclin dependent kinase 1
20	1020	CDK5	cyclin dependent kinase 5
21	1129	CHRM2	cholinergic receptor muscarinic 2
22	1131	CHRM3	cholinergic receptor muscarinic 3
23	1137	CHRNA4	cholinergic receptor nicotinic alpha 4 subunit
24	1141	CHRNB2	cholinergic receptor nicotinic beta 2 subunit
25	1436	CSF1R	colony stimulating factor 1 receptor
26	3579	CXCR2	C-X-C motif chemokine receptor 2
27	1565	CYP2D6	cytochrome P450 family 2 subfamily D member 6
28	1812	DRD1	dopamine receptor D1
29	1813	DRD2	dopamine receptor D2
30	1814	DRD3	dopamine receptor D3
31	1815	DRD4	dopamine receptor D4
32	1859	DYRK1A	dual specificity tyrosine phosphorylation regulated kinase 1A
33	1889	ECE1	endothelin converting enzyme 1
34	9451	EIF2AK3	eukaryotic translation initiation factor 2 alpha kinase 3
35	2064	ERBB2	erb-b2 receptor tyrosine kinase 2
36	2078	ERG	ETS transcription factor ERG
37	2260	FGFR1	fibroblast growth factor receptor 1
38	2261	FGFR3	fibroblast growth factor receptor 3
39	2534	FYN	FYN proto-oncogene, Src family tyrosine kinase
40	2558	GABRA5	gamma-aminobutyric acid type A receptor subunit alpha5
41	51738	GHRL	ghrelin and obestatin prepropeptide
42	2904	GRIN2B	glutamate ionotropic receptor NMDA type subunit 2B
43	2932	GSK3B	glycogen synthase kinase 3 beta
44	3091	HIF1A	hypoxia inducible factor 1 subunit alpha
45	3290	HSD11B1	hydroxysteroid 11-beta dehydrogenase 1
46	3350	HTR1A	5-hydroxytryptamine receptor 1A
47	3356	HTR2A	5-hydroxytryptamine receptor 2A
48	3358	HTR2C	5-hydroxytryptamine receptor 2C
49	3360	HTR4	5-hydroxytryptamine receptor 4
50	3362	HTR6	5-hydroxytryptamine receptor 6
51	3363	HTR7	5-hydroxytryptamine receptor 7
52	3480	IGF1R	insulin like growth factor 1 receptor
53	3643	INSR	insulin receptor
54	3832	KIF11	kinesin family member 11
55	3815	KIT	KIT proto-oncogene, receptor tyrosine kinase
56	3932	LCK	LCK proto-oncogene, Src family tyrosine kinase
57	120892	LRRK2	leucine rich repeat kinase 2
58	4128	MAOA	monoamine oxidase A
59	5602	MAPK10	mitogen-activated protein kinase 10
60	10461	MERTK	MER proto-oncogene, tyrosine kinase
61	4233	MET	MET proto-oncogene, receptor tyrosine kinase
62	4311	MME	membrane metalloendopeptidase
63	4313	MMP2	matrix metallopeptidase 2
64	4314	MMP3	matrix metallopeptidase 3
65	4318	MMP9	matrix metallopeptidase 9
66	2475	MTOR	mechanistic target of rapamycin kinase
67	23385	NCSTN	nicastrin
68	4842	NOS1	nitric oxide synthase 1
69	4843	NOS2	nitric oxide synthase 2
70	4846	NOS3	nitric oxide synthase 3
71	4835	NQO2	N-ribosyldihydronicotinamide:quinone reductase 2
72	4914	NTRK1	neurotrophic receptor tyrosine kinase 1
73	4915	NTRK2	neurotrophic receptor tyrosine kinase 2
74	4916	NTRK3	neurotrophic receptor tyrosine kinase 3
75	5027	P2RX7	purinergic receptor P2X 7
76	142	PARP1	poly(ADP-ribose) polymerase 1
77	5144	PDE4D	phosphodiesterase 4D
78	8622	PDE8B	phosphodiesterase 8B
79	5159	PDGFRB	platelet derived growth factor receptor beta
80	7941	PLA2G7	phospholipase A2 group VII
81	5663	PSEN1	presenilin 1
82	5664	PSEN2	presenilin 2
83	55851	PSENEN	presenilin enhancer, gamma-secretase subunit
84	5743	PTGS2	prostaglandin-endoperoxide synthase 2
85	6571	SLC18A2	solute carrier family 18 member A2
86	6531	SLC6A3	solute carrier family 6 member 3
87	6532	SLC6A4	solute carrier family 6 member 4
88	6752	SSTR2	somatostatin receptor 2
89	6753	SSTR3	somatostatin receptor 3
90	6755	SSTR5	somatostatin receptor 5

**Table 3:** Top 20 KEGG pathway terms enriched in UR alkaloids against AD.

Term	Pathway	Rich Factor	*p*-Value	Count	Symbols
hsa05010	Alzheimer disease	0.08	2.445 × 10^−18^	16	CAPN1,CASP3,CDK5,GRIN2B,GSK3B,MME,NOS1,PSEN1,PSEN2,PTGS2,ADAM17,EIF2AK3,NCSTN,BACE1,PSENEN,APH1B
hsa04726	Serotonergic synapse	0.11	9.421 × 10^−17^	13	ALOX5,CASP3,CYP2D6,HTR1A,HTR2A,HTR2C,HTR4,HTR6,HTR7,MAOA,PTGS2,SLC6A4,SLC18A2
hsa04020	Calcium signaling pathway	0.07	1.693 × 10^−16^	15	ADRB2,CHRM2,CHRM3,DRD1,ERBB2,HTR2A,HTR2C,HTR4,HTR6,HTR7,NOS1,NOS2,NOS3,P2RX7,PDGFRB
hsa04151	PI3K-Akt signaling pathway	0.04	4.79 × 10^−15^	17	AKT1,CCND1,CHRM2,CSF1R,ERBB2,FGFR1,FGFR3,MTOR,GSK3B,IGF1R,INSR,KIT,MET,NOS3,NTRK1,NTRK2,PDGFRB
hsa04728	Dopaminergic synapse	0.08	5.086 × 10^−13^	11	AKT1,DRD1,DRD2,DRD3,DRD4,GRIN2B,GSK3B,MAOA,MAPK10,SLC6A3,SLC18A2
hsa04722	Neurotrophin signaling pathway	0.07	2.555 × 10^−10^	9	ABL1,AKT1,GSK3B,NTRK1,NTRK2,NTRK3,MAPK10,PSEN1,PSEN2
hsa04330	Notch signaling pathway	0.12	1.685 × 10^−8^	6	PSEN1,PSEN2,ADAM17,NCSTN,PSENEN,APH1B
hsa04725	Cholinergic synapse	0.06	9.258 × 10^−8^	7	ACHE,AKT1,CHRM2,CHRM3,CHRNA4,CHRNB2,FYN
hsa04144	Endocytosis	0.03	2.33 × 10^−7^	9	ADRB2,CCR5,CSF1R,FGFR3,IGF1R,CXCR2,KIT,MET,NTRK1
hsa04520	Adherens junction	0.07	2.494 × 10^−7^	6	ERBB2,FGFR1,FYN,IGF1R,INSR,MET
hsa04926	Relaxin signaling pathway	0.05	2.92 × 10^−7^	7	AKT1,MMP2,MMP9,NOS1,NOS2,NOS3,MAPK10
hsa04540	Gap junction	0.07	4.675 × 10^−7^	6	CDK1,DRD1,DRD2,HTR2A,HTR2C,PDGFRB
hsa04210	Apoptosis	0.05	5.935 × 10^−7^	7	PARP1,AKT1,CAPN1,CASP3,NTRK1,MAPK10,EIF2AK3
hsa04012	ErbB signaling pathway	0.06	6.051 × 10^−7^	6	ABL1,AKT1,ERBB2,MTOR,GSK3B,MAPK10
hsa04657	IL-17 signaling pathway	0.06	6.442 × 10^−7^	6	CASP3,GSK3B,MMP3,MMP9,MAPK10,PTGS2
hsa04371	Apelin signaling pathway	0.04	7.882 × 10^−6^	6	AKT1,CCND1,MTOR,NOS1,NOS2,NOS3
hsa05014	Amyotrophic lateral sclerosis	0.07	3.464 × 10^−5^	4	CASP1,CASP3,GRIN2B,NOS1
hsa05032	Morphine addiction	0.04	0.0002249	4	DRD1,GABRA5,PDE4D,PDE8B
hsa04360	Axon guidance	0.03	0.0004339	5	ABL1,CDK5,FYN,GSK3B,MET
hsa04115	p53 signaling pathway	0.04	0.00213	3	CCND1,CASP3,CDK1

**Table 4:** Molecular docking of core targets with corynoxine and corynantheine.

Ligands	Target	PDB	deltaG (kcal/mol)	deltaGvdw	FullFitness (kcal/mol)	Energy (kcal/mol)
Corynoxine	CCR5	6AKY	−9.28	−83.41	−1260.02	16.61
Corynoxine	MMP3	2D1O	−8.52	−70.64	−866.94	16.06
Corynoxine	CCND1	2W9F	−8.17	−66.74	−1571.13	21.40
Corynoxine	NTRK2	4AT4	−8.10	−56.35	−1692.15	1.58
Corynoxine	GRIN2B	7KL4	−7.95	−76.52	−1938.77	29.92
Corynoxine	CHRM2	4MQT	−7.92	−51.24	−874.59	14.59
Corynoxine	CDK5	1UNH	−7.90	−54.59	−3964.05	20.41
Corynoxine	MMP2	1QIB	−7.67	−54.18	−1258.85	16.61
Corynoxine	GSK3B	2O5K	−7.66	−62.37	−1986.98	17.42
Corynantheine	CHRM2	4MQT	−9.38	−82.53	−860.06	−5.88
Corynantheine	MMP3	2D1O	−9.30	−82.62	−857.66	−0.88
Corynantheine	MMP2	1QIB	−9.24	−94.70	−1246.61	−5.34
Corynantheine	GRIN2B	7KL4	−9.02	−87.46	−1941.76	−5.64
Corynantheine	CCR5	6AKY	−8.92	−75.97	−1250.69	1.62
Corynantheine	CCND1	2W9F	−8.77	−83.19	−1556.05	8.76
Corynantheine	NTRK2	4AT4	−8.10	−53.43	−1676.61	−3.78
Corynantheine	CDK5	1UNH	−7.95	−61.20	−3952.57	−1.90
Corynantheine	GSK3B	2O5K	−7.38	−56.22	−1952.87	24.20

## Data Availability

Processed data is contained within the article. Raw data is available from the corresponding author upon request.

## Abbreviations

AD	Alzheimer’s disease
APH1B	Aph-1 homolog b
APP	β-amyloid precursor protein
Aβ	amyloid beta
BCHE	Butyrylcholinesterase
BP	Biological process
CYP2D6	Cytochrome p450 family 2 subfamily d member 6
GEO	Gene Expression Omnibus
GO	Gene Ontology
Hacc	Hydrogen bond acceptors
Hdon	Hydrogen bond donors
HPLC	High-performance liquid chromatography
KEGG	Kyoto Encyclopedia of Genes and Genomes
MCODE	Molecular complex detection
MET	Met proto-oncogene receptor tyrosine kinase
MW	Molecule weight
NCT	Nicastrin
NFT	Neurofibrillary tangles
N-methyl D-aspartate	Nmda
NTRK1	Neurotrophic receptor tyrosine kinase 1
PPI	Protein–protein interaction
Rbon	Rotatable bonds
SP	Senile plaques
SSTR3	Somatostatin receptor 3
TCMSP	Traditional Chinese Medicine Systems Pharmacology Database and Analysis Platform
TPSA	Topological polar surface area
TTD	Therapeutic target database
UR	*Uncaria rhynchophylla*
